# Comparative renal physiology and excretory adaptations in ruminants and poultry: Implications for nutrition, health, and environmental management

**DOI:** 10.1016/j.psj.2026.107007

**Published:** 2026-04-23

**Authors:** Dawudie Gobezie

**Affiliations:** Department of Animal Science, College of Veterinary Medicine and Animal Sciences, Samara University, P.O. Box: 132, Samara, Ethiopia

**Keywords:** Poultry, Physiology, Urea, Environmental emission, Ruminant

## Abstract

The urinary systems of ruminants and poultry represent an evolutionary adaptation to distinct ecological niches, digestive strategies, and water conservation needs. This *critical review* employs a systematic approach to compare these systems, analyzing their anatomical, physiological, and functional divergences with emphasis on contemporary agricultural challenges. Ruminants, as ureotelic mammals, possess a urinary system where nitrogen excretion is dominated by urea, heavily influenced by rumen microbial metabolism and the urea nitrogen recycling loop. This physiology underpins both the use of urinary purine derivatives as markers for microbial protein synthesis and the significant environmental burden of nitrous oxide (N₂O) emissions from urine patches. In contrast, the avian uricotelic system, characterized by a mixed population of loopless (reptilian type) and looped (mammalian type) nephrons and a cloacal terminus, prioritizes water conservation and eggshell calcification but presents unique pathologies like gout. By synthesizing scattered literature, this review provides a novel, integrated framework linking core physiology to applied outcomes in nutrition, disease management, and environmental sustainability. This review identified critical knowledge gaps, particularly in avian renal microanatomy and the precise modeling of dietary interventions on nitrogen loss and proposes specific, actionable research directions to advance sustainable animal production.

## Highlights

• **This manuscript** provides a detailed synthesis of the comparative physiology and functional adaptations of the urinary system in ruminants and poultry.
• Highlights the fundamental physiological difference between ruminants primarily excrete nitrogen as urea while birds excrete it as uric acid.
• Points to the need for more detailed histological studies of avian kidneys and integrated research linking ruminant nutrition directly to on-farm emission metrics.

## Introduction

Homeostasis in terrestrial vertebrates is critically maintained by the urinary system, an organ complex responsible for waste excretion, electrolyte and acid-base balance, and hormonal regulation (Sherwood et al., 2005). Beyond these conserved functions, the urinary system exhibits remarkable adaptive diversity that reflects phylogenetic history, diet, and habitat. In livestock species, understanding this diversity is not merely academic; it is fundamental to optimizing animal health, nutritional efficiency, and mitigating environmental impacts (Pinares-Patiño et al., 2007).

Ruminants (cattle, sheep, and goats) and poultry (chickens, turkeys) constitute the cornerstone of global animal protein production yet embody two profoundly different physiological paradigms. Ruminants are foregut fermenters with a nitrogen economy revolving around microbial protein synthesis in the rumen. Their excretory endpoint is primarily urea, a highly soluble compound whose soil deposition drives reactive nitrogen losses (Oenema et al., 2008). Poultry, as birds, are uricotelic, synthesizing insoluble uric acid as a water-saving adaptation crucial for flight and arid environments (Braun and Wideman, 2015). Their renal anatomy lacks a discrete bladder, and nephron structure differs significantly from the mammalian model. The avian kidneys contain two-nephron types (reptilian-type and mammalian-type nephrons). The fundamental distinction between reptilian-type and mammalian-type nephrons lies in the presence of a loop of Henle and the consequent capacity for urinary concentration. Reptilian-type nephrons lack a loop of Henle entirely, with a simple tubular architecture that limits their ability to produce urine hyperosmotic to plasma; in contrast, mammalian-type nephrons possess a well-developed, U-shaped loop of Henle that, together with a countercurrent multiplier system and a prominent renal medulla, enables the generation of highly hyperosmotic urine (Al-Agele., 2024; Braun and Wideman, 2015). A common misconception holds that avian nephrons lack a loop of Henle. In reality, the avian kidney contains both types: most nephrons are reptilian-type without a loop, but minorities are mammalian-type with a short loop of Henle. These avian loops are anatomically present but functionally weak, explaining why birds do not produce highly concentrated urine despite possessing the structure (Braun and Wideman, 2015).

Despite their economic importance, a direct, critical synthesis comparing these systems is currently lacking. Most reviews focus on a single species or class, and research in this area remains notably siloed: recent work on ruminant urinary health is confined to species-specific pathologies such as obstructive urolithiasis, while contemporary avian studies concentrate exclusively on the histological adaptations of the renal-cloacal axis in domestic fowl (Adhikari and Shah, 2025; Saritha, 2024). Notably, recent comparative reviews have successfully bridged the gap between ruminants and poultry with respect to digestive physiology, encompassing both gastrointestinal microbiota composition and enzymatic digestive efficiency (Kuzmina et al., 2024; Tardiolo et al., 2025). However, a parallel synthesis addressing the divergent urinary mechanisms of these two economically critical classes is still absent from the literature. This gap impedes a holistic understanding of how fundamental physiology dictates responses to nutritional strategies, disease susceptibility and manure management. Therefore, this review aims to: 1) Systematically compare the anatomy and physiology of the urinary system in ruminants and poultry; 2) Critically analyze the pathway from diet to excretory product, with emphasis on quantifying nitrogen excretion rates and modeling environmental footprints; 3) Evaluate species-specific health challenges related to urinary function; and 4) Synthesize practical applications and future research priorities for sustainable production. A systematic methodology underpins this comparative analysis to ensure comprehensiveness and reproducibility.

## Materials and method

To ensure a rigorous and transparent synthesis, this review was conducted following a structured, systematic approach.

### Literature search strategy

Electronic databases (PubMed, Scopus, Google Scholar, and Web of Science) were searched. To ensure comprehensive coverage and avoid the inadvertent exclusion of species‑specific studies, two parallel search strings were executed independently: one for ruminant domain and another for poultry domain, after which comparative results were combined. A combination of keywords and Boolean operators was used:

**Ruminant search:** ("urinary system" OR kidney OR nephron OR "renal physiology") AND ("ruminant*" OR cattle OR sheep OR goat OR bovine OR ovine) AND ("nitrogen excretion" OR urea OR "purine derivative*" OR "environmental emission*" OR "water balance").

• **Poultry search:** ("urinary system" OR kidney OR nephron OR "renal physiology") AND ("poultry" OR chicken OR avian OR fowl OR gallus) AND ("nitrogen excretion" OR "uric acid" OR "purine derivative*" OR "environmental emission*" OR "water balance").

### Screening and selection criteria

Titles and abstracts were screened for relevance to urinary/anatomy/physiology/nitrogen metabolism in the target species. Full-text articles of potentially relevant studies were obtained and assessed based on the following inclusion criteria: (1) primary research or authoritative reviews on ruminant or avian urinary system structure or function; (2) studies investigating diet effects on urine composition; (3) research on environmental impacts of urinary nitrogen; (4) studies on relevant pathophysiology specifically urinary tract infections, urolithiasis, gout, and nephrotoxicity. Exclusion criteria included non-English language articles without available translation and articles where urinary data were not the primary focus. Of the 668 records screened by title and abstract, 37 (5.5%) were excluded solely due to language inaccessibility (non‑English without translation).

### Data extraction and synthesis

**Fig. 1:**
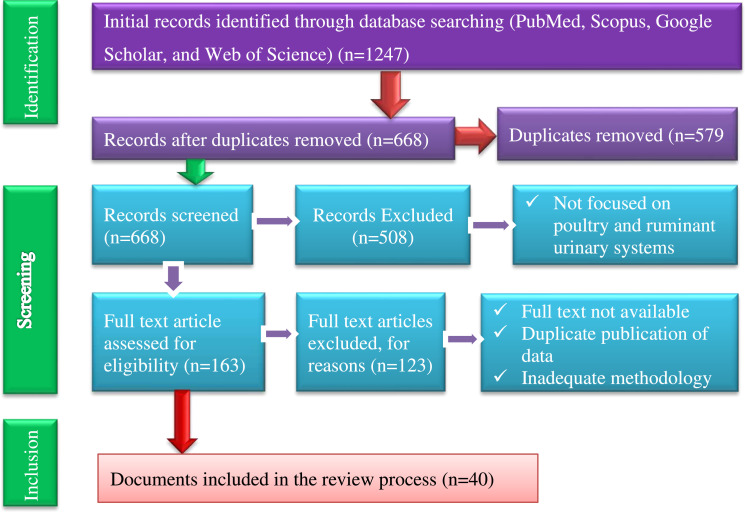
Flow diagram of the systematic literature search and study selection process employed in this review.

**Fig. 2:**
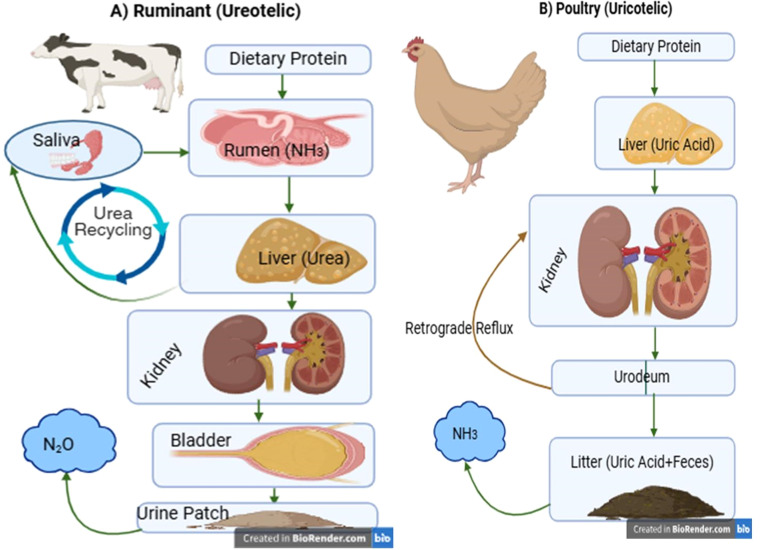
Schematic comparison of nitrogen excretion pathways in (A) ruminants and (B) poultry. Panel A (ureotelic system): Dietary protein is fermented in the rumen to ammonia (NH₃), which is converted to urea in the liver. Urea undergoes either renal excretion via the bladder and urethra or recycling back to the rumen via saliva (curved arrow). Urinary urea deposited in soil drives microbial N₂O emissions. Panel B (uricotelic system): Dietary protein is metabolized to uric acid in the liver, secreted by the kidneys, and excreted directly into the urodeum of the cloaca. Retrograde reflux from the cloaca into the hindgut enables further water resorption. Uric acid in mixed excreta is mineralized by microbial urease in litter, releasing NH₃. Based on physiological principles reviewed in Braun and Wideman (2015); Lapierre and Lobley (2001), and Braun (1999); Huntington & Archibeque (1999); Miles et al. (2011); Pinares-Patiño et al. (2007).

Data on species, anatomical descriptions, physiological metrics (urine concentration, nitrogen partitioning, and excretion rates), dietary influences, and environmental impact factors were extracted into a standardized matrix. Information was synthesized thematically according to a predefined comparative framework (Anatomy, Physiology, Health, and Environment). Critical analysis involved identifying consensus, contradictions and knowledge gaps across the literature Fig. 1, 2.

### Literature search outcomes

• The combined search strategy (ruminant and poultry search) yielded 1247 initial records. Following the removal of 579 duplicate entries, 668 unique records were screened based on title and abstract. Of these, 508 were excluded for failure to meet the predefined inclusion criteria. The remaining 163 full-text articles were assessed for eligibility, resulting in the inclusion of 40 studies in the final synthesis. The 123 excluded articles were omitted for the following reasons: insufficient methodological detail (*n* = 74), duplicate publication of data (*n* = 33), or unavailability of the full text (*n* = 16).

## Foundational divergence: a comparative anatomical overview

The structural differences between the ruminant and avian urinary systems are profound, each optimized for distinct physiological priorities (Dyce et al., 2002; King and McLelland, 1984).

### The ruminant system: a ureotelic architecture

The ruminant system follows the typical mammalian plan: paired multilobar kidneys (with a distinctive external lobulation in bovine species), ureters, a muscular urinary bladder, and a urethra. In bovines, the renal pelvis is extensively branched, forming prominent recesses (calices) that surround each medullary pyramid and coalesce into major calices before draining into the ureter. This specialized pelvic architecture facilitates efficient urine collection and flow from the multilobar parenchyma, reducing stasis and potential for ascending infection (Dyce et al., 2002). A notable feature in the female bovine and buffalo is the suburethral diverticulum, which can complicate catheterization (Habel, 1975). The nephron population includes both cortical and juxtamedullary nephrons with loops of Henle of varying lengths, enabling the production of urine hyperosmotic to plasma a key adaptation for water conservation, though less efficient than in some desert-adapted mammals (Skadhauge, 1981).

### The avian system: a cloacal-terminated, uricotelic design

Avian anatomy deviates radically. Paired kidneys are deeply embedded within the synsacrum, typically divided into cranial, middle, and caudal lobes. They are drained by ureters that empty directly into the urodeum of the cloaca; a urinary bladder is absent (King and McLelland, 1984). The nephron population is heterogeneous. The majority are reptilian‑type nephrons, which lack a loop of Henle and are confined to the cortical region (Braun and Wideman, 2015). In domestic fowl (Gallus gallus), approximately 75-80% of nephrons are of this loopless type (Johnson and Mugaas, 1970), though the proportion ranges from 70 to 90% across avian orders (Braun, 1999). A minority are mammalian-type nephrons, possessing a loop of Henle that penetrates into medullary cones, allowing for some urinary concentrating ability (Braun, 1999). This two-nephron system represents a functional compromise Table 1.

**Table 1:** Comparative anatomy and physiology of the urinary systems of ruminants and poultry.

Feature	Ruminants	Poultry	Key Citations
Primary Nitrogenous Waste	Urea (water-soluble)	Uric acid (insoluble colloid/paste)	Braun and Wideman, 2015
System Terminology	Ureotelic	Uricotelic	Sherwood et al., 2005
Water Conservation Priority	Moderate	High (adaptation for flight, arid environments, and eggshell formation)	Braun and Wideman, 2015
Urinary Bladder	Present	Absent	Dyce et al., 2002;
Final Excretion Route	Urethra	Cloaca (via ureters into urodeum)	Dyce et al., 2002
Kidney Morphology	Multilobar with external lobulation (bovine); and renal pelvis	Paired, elongated, divided into cranial, middle, and caudal lobes; embedded in synsacrum	Dyce et al., 2002; King and McLelland, 1984
Nephron Types	Juxtamedullary & cortical nephrons with Loops of Henle	Heterogeneous: Mammalian-type (with loop) & Reptilian-type (loopless)	Braun and Wideman, 2015
Urine Form	Liquid, often copious volume	Semi-solid, mixed with feces as "droppings"	Goldstein and Skadhauge, 2000
Key Nitrogen Recycling Mechanism	Urea nitrogen recycling via saliva and rumen wall (rumen-liver-kidney axis)	Retrograde cloacal reflux of urine into colon/ceca for post-renal water and electrolyte resorption	Braun, 1999; Lapierre and Lobley, 2001
Major Environmental Concern	N₂O from soil urea hydrolysis (urine patches)	NH₃ volatilization from litter (uric acid mineralization)	Miles et al., 2011; Pinares-Patiño et al., 2007
Primary Health Disorders	Obstructive urolithiasis, pyelonephritis, cystitis	Visceral and articular gout, urolithiasis	Kshetriyamayum., 2026; Radositis et al., 2007; Wideman, 2016

## Core physiology: from diet to excretory product

The transformation of dietary nitrogen into excretory waste highlights the deepest functional divide between these groups.

### The ruminant: a system dominated by rumen-kidney-liver axis

Ruminant nitrogen metabolism is a tripartite dialogue between the rumen, liver, and kidneys. Dietary protein is extensively degraded to ammonia by rumen microbes. This ammonia is either incorporated into microbial protein or absorbed into the portal blood. The liver detoxifies this ammonia via the urea cycle (Lapierre and Lobley, 2001). The resulting urea has two fates: 1) excretion via the kidneys, or 2) recycling back to the rumen via saliva and direct rumen wall transfer, providing nitrogen for microbial growth (Huntington and Archibeque, 1999). This recycling is a critical efficiency mechanism but also means urinary urea output is highly sensitive to dietary protein balance. Excess dietary protein leads to a linear increase in urinary urea nitrogen, a key driver of environmental loss (Tamminga, 1992).

Urinary purine derivatives (allantoin, uric acid, xanthine, hypoxanthine) originate almost exclusively from the catabolism of microbial nucleic acids absorbed from the gut. Their excretion rate is correlated with microbial protein flow to the duodenum, forming the basis of a non-invasive method for estimating microbial protein synthesis (Chen and Gomes, 1992). Notably, the profile differs between cattle (predominantly allantoin) and sheep (all four PDs), reflecting differences in post-absorptive metabolism (Gonzalez-Ronquillo et al., 2003).

### The avian: uricotelism and integrated cloacal function

Birds synthesize uric acid in the liver via a complex, adenosine triphosphate (ATP)-dependent pathway. Its low solubility allows it to be excreted as a semi-solid paste with minimal water loss, a paramount adaptation (Goldstein and Skadhauge, 2000). Uric acid is secreted by renal tubules (not just filtered) and travels via the ureters to the cloaca.

The cloaca performs a crucial post-renal modification. Through retrograde peristalsis, urine can be moved back into the colon and ceca, where microbial action and further water resorption can occur before final excretion (Braun, 1999). This renal-cloacal axis is unique to birds and reptiles. Furthermore, the avian kidney plays a vital role in calcium metabolism for eggshell formation. During lay, a significant portion of blood calcium is bound to phosphorus-rich vitellogenin. The kidney must efficiently excrete the liberated phosphate load while conserving calcium, a delicate balance that can be disrupted by mineral imbalances (Proszkowiec-Weglarz and Angel, 2013).

## Health implications: contrasting pathophysiologies

The structural and metabolic differences predispose each group to distinct urinary disorders.

### Ruminant health challenges

The primary concerns are infectious and obstructive. The relatively long urethra, especially in males, and the presence of a bladder create a system susceptible to ascending bacterial infections (cystitis) and obstructive urolithiasis. Urolithiasis, often associated with high-grain diets altering urine pH and mineral composition, is a major cause of morbidity in feedlot steers and lambs (Radositis et al., 2007). Pyelonephritis is a clinically significant condition in cattle, especially in postpartum dairy cows, where ascending infection from the lower urinary tract can lead to severe renal parenchymal damage. Urinary tract affections in sheep are less commonly reported in the literature compared to cattle, and pyelonephritis is considered primarily a bovine disease, with ovine cases occurring only occasionally (Jyoti et al., 2012).

### Avian health challenges

The hallmark urinary disorder in poultry is visceral and articular gout. This occurs when uric acid levels in the blood (hyperuricemia) exceed the renal secretory capacity, leading to precipitation of urate crystals in visceral surfaces and joints (Wideman, 2016). Etiologies are multifactorial, including: 1) Renal damage from nephrotoxins (e.g., certain mycotoxins, antibiotics, high dietary calcium); 2) Dehydration reducing urinary water flow; and 3) Metabolic alkalosis, which decreases uric acid solubility. Quantitatively, dietary calcium levels exceeding 4-5% are associated with a marked increase in the incidence of visceral gout in broiler chickens (Selle et al., 2019). Elevated dietary calcium promotes the formation of insoluble calcium–urate complexes, impairs renal clearance, and exacerbates hyperuricemia. Susceptibility also varies by breed and production system: fast‑growing broiler lines are at heightened risk due to their elevated metabolic rates and rapid skeletal mineralization, while certain genetic lines (e.g., Leghorns and Cornish crosses) show increased predisposition (Kshetriyamayum, 2026). Laying hens during peak production face additional challenges from the intense calcium flux required for eggshell formation (Wideman, 2016). The absence of a urinary bladder further compounds the problem, as any impairment of renal function leads to rapid systemic accumulation of uric acid and swift clinical deterioration.

## Environmental and practical applications: from physiology to management

### The ruminant nitrogen emission challenge

Urinary nitrogen, primarily as urea, is the most volatile and leachable fraction of manure. Upon deposition on soil, urea is rapidly hydrolyzed by urease enzymes to ammonium (NH₄⁺), which is then subject to nitrification (to nitrate, NO₃⁻) and denitrification (to N₂O and N₂). N₂O is a potent greenhouse gas (Pinares-Patiño et al., 2007). Mitigation strategies are directly rooted in physiology: 1) Precision protein nutrition to reduce excess nitrogen intake (Kebreab et al., 2010); 2) Dietary additives like nitrification inhibitors that slow the soil conversion of ammonium to nitrate; 3) Increasing urine volume through salt supplementation to dilute urea concentration (Spek et al., 2012).

### Poultry litter management

Poultry excreta’s, being a mixture of uric acid and feces, is typically managed as solid "litter." Upon exposure to moisture and microbial urease, uric acid is mineralized to urea and then to ammonia (NH₃), leading to significant aerial ammonia emissions within housing, a welfare and environmental concern (Miles et al., 2011). Ammonia emission rates from broiler houses typically range from 0.2 to 0.5 kg NH₃ per bird per year, depending on ventilation, litter management, and dietary protein content (Miles et al., 2011; Moore et al., 2000). Moore et al. (2011) further estimated that approximately 46 g of NH₃ is emitted per broiler chicken over a single grow-out period. Elevated in-house NH₃ concentrations exceeding 25 ppm are associated with reduced feed intake, impaired growth, respiratory irritation, and increased susceptibility to disease (Miles et al., 2011; Wang et al., 2022). Furthermore, the high phosphorus content of poultry litter relative to crop needs can lead to phosphorus runoff and eutrophication of surface water. Phosphorus (p) in poultry manure exists in both organic (phytate‑bound) and inorganic forms. Dietary inclusion of exogenous phytase enzymes enhances the hydrolysis of phytate‑P in the gastrointestinal tract, increasing P retention by 20–30% and proportionally reducing total P excretion in manure (Angel et al., 2005; Selle and Ravindran, 2007).

Mitigation strategies extend beyond nutrition to include litter amendments. Acidifying agents such as aluminum sulfate (alum) reduce litter pH, thereby inhibiting urease activity and NH₃ volatilization by 30-50% (Moore et al., 2000). Similarly, sodium bisulfate applied at rates of 2-7% (w/w) has been shown to reduce NH₃ emissions by 90-100% under controlled conditions (Poudel et al., 2024). Zeolites, microporous aluminosilicate minerals, adsorb ammonium ions via cation exchange and reduce aerial NH₃ concentrations in poultry houses (Li et al., 2008); recent work indicates that zeolite inclusion at 8-11% (w/w) in litter reduces NH₃ emissions by 20-33% (Poudel et al., 2024). A novel zeolite‑clay‑rice husk ash composite bedding applied at 40% of rice husk weight lowered in‑house NH₃ concentrations by approximately 50% while simultaneously improving footpad health and reducing physiological stress markers (Nguyen et al., 2026). Biochar amendments at 13-17% (w/w) also achieve substantial NH₃ reductions of 41-46% (Poudel et al., 2024). A recent meta‑analysis confirmed that both acidifiers and adsorbents are effective in lowering NH₃ emissions from poultry litter (Rehman et al., 2021). Nutritional strategies, such as using phytase enzymes to improve phosphorus digestibility and formulating diets with reduced protein and balanced amino acids, are critical for minimizing excretion (Angel et al., 2005).

### Resource recovery potential

Both waste streams have valorization potential. Ruminant slurry is a valuable liquid fertilizer if applied accurately. Poultry litter is used as fertilizer, in composting, or as a feedstock for anaerobic digestion and bioenergy, though its high nitrogen content requires careful process management to avoid ammonia inhibition (Eduok et al., 2018).

## Future perspectives and knowledge gaps

This comparative analysis highlights several critical knowledge gaps that should guide future research. First, the functional plasticity of the avian two-nephron system under modern production stressors (e.g., heat stress, high-density rearing) remains poorly characterized. Advanced imaging modalities such as micro-computed tomography could map three-dimensional nephron architecture and perfusion in commercial broiler lines. Complementary single-cell transcriptomic approaches would clarify nephron-specific gene expression patterns. Second, integrated farm-scale models are needed that dynamically couple mechanistic rumen fermentation algorithms (e.g., the Molly model) with poultry litter emission equations. Such models would enable systems-level comparisons of nitrogen use efficiency and environmental trade-offs in mixed farming operations.

Third, the host–microbiome-kidney axis warrants deeper investigation. In ruminants, multi-omics approaches could identify microbial signatures linked to enhanced urea recycling and nitrogen retention. In poultry, the cecal microbiome's role in post-renal modification of uric acid composition remains underexplored. Elucidating how hindgut fermentation alters the nitrogenous profile of excreta before litter contact could reveal novel probiotic or prebiotic strategies for reducing in-house ammonia generation. Fourth, no preclinical diagnostic tools currently exist for avian gout. Identifying early metabolomic or proteomic biomarkers of impending hyperuricemia would substantially improve flock health management and reduce economic losses. Finally, advancing valorization technologies for nitrogen- and phosphorus-rich manures is essential. Approaches such as struvite precipitation and ammonia stripping can transform waste streams into renewable resources, closing nutrient loops in sustainable poultry and ruminant production systems.

## Conclusion

This critical analysis reveals that the fundamental divergence between the ruminant's ureotelic system and the avian uricotelic system is a powerful determinant of nutrition, health, and environmental impact. The ruminant kidney, as a partner to rumen fermentation, creates a trade-off between nitrogen efficiency and significant greenhouse gas potential. In contrast, the avian kidney-cloaca axis prioritizes water conservation at a high metabolic cost, predisposing to unique pathologies like gout. Bridging this physiological understanding to applied strategies through precision nutrition, targeted health interventions, and innovative waste management is essential for sustainable intensification. Future research must therefore pivot from descriptive studies to integrative solutions including advanced renal imaging and coupled digestive-excretory modeling that simultaneously optimize animal physiology and environmental stewardship.

## Ethics statement

Ethics approval was not required for this research.

## Data availability statement

Not applicable

## Disclosures

The author declares that there is no conflict of interest.
